# Implementing a Mind-Body Skills Group in Psychiatric Residency Training

**DOI:** 10.1007/s40596-021-01507-x

**Published:** 2021-08-02

**Authors:** Noshene Ranjbar, Matt Erb, Julie Tomkins, Krishna Taneja, Amelia Villagomez

**Affiliations:** 1 University of Arizona, Tucson, AZ USA; 2 The Center for Mind-Body Medicine, Washington, DC USA; 3 Southern Illinois University, Springfield, IL USA

**Keywords:** Resiliency, Resident well-being, Mind-body medicine, Burnout

## Abstract

**Objective:**

The burnout crisis in healthcare has led to interventions promoting resiliency and wellness among residents. One such intervention is a 10-week Mind-Body Skills Group including didactics and experiential exercises, self-expression, and small-group support. A Mind-Body Skills Group for residents and fellows in the University of Arizona-Tucson Department of Psychiatry aimed to teach skills for self-care and patient care.

**Methods:**

In 2018–2020, 50 University of Arizona-Tucson psychiatric residents and fellows participated in Mind-Body Skills Groups. After finishing the course, 44 participants completed a survey about satisfaction with the course and its content, comfort sharing experiences with the group, use of mind-body skills for participants’ own self-care and wellness, use of these skills with patients, and likelihood of recommending the Mind-Body Skills Group to colleagues.

**Results:**

Eighty-four percent of survey respondents were satisfied or very satisfied with the Mind-Body Skills Group. Eighty percent used skills learned in the course for their own self-care and 61% used the skills with patients on at least a weekly basis. Eighty-nine percent indicated they were likely or very likely to use mind-body skills with patients in the future. Ninety-five percent of respondents felt safe sharing personal feelings and experiences in the group, and 95% would strongly recommend or likely recommend the course to colleagues. Results for in-person and online groups were not significantly different.

**Conclusions:**

A 10-week Mind-Body Skills Group during psychiatric residency was well received by participants. The course influenced personal health behaviors, which may bolster resiliency and reduce risk for burnout.

Burnout, which arises from occupational stressors, is very high among US physicians [1], with an estimated prevalence of 42% in 2020 [2]. Burnout takes a toll on individual physicians and their families, places a strain on the healthcare system, and adversely affects the quality of patient care [3, 4]. Training physicians, medical students, residents, and fellows in mind-body skills or mindfulness can promote resiliency, which may help to reduce burnout. Research indicates that positive effects of such training may arise through the mitigation of toxic stress and improvement in active coping skills [1, 5, 6].

Recent publications indicate that healthcare institutions have begun to adopt courses and programs that promote resilience in healthcare professionals [6–11]. As the body of research on interventions to combat burnout grows, it is important to assess multiple aspects of implementing such courses in addition to measuring effects on burnout. Aspects that should be evaluated include acceptability and reception by participants, how these interventions influence participants’ personal behavior, and how they influence care of patients.

This paper details results of a study at the University of Arizona College of Medicine-Tucson Psychiatry Department (UAPD), in which three cohorts of residents and fellows were surveyed after their participation in 10-week Mind-Body Skills Groups from 2018 to 2020. The Mind-Body Skills Group model was chosen because of its previously documented utility for healthcare settings [6, 12, 13]. This article discusses overall participant ratings of the Mind-Body Skills Group at the UAPD, how frequently participants used the techniques taught for their own self-care, and how often they used or expected to use these techniques with patients in the future.

## Methods

The Mind-Body Skills Group was originally implemented in 2015 at the UAPD as part of the Integrative Psychiatry Elective Track, which consists of three main components: a series of interactive online modules providing relevant medical knowledge and evidential support for integrative healthcare methods; a supervised clinical component allowing residents and fellows to critically discern the appropriateness of integrative health approaches as they apply to clinical psychiatric care; and the Mind-Body Skills Group, a self-care component that facilitates hands-on practice with techniques to provide an experiential foundation in mind-body skills [14]. This paper concerns the Mind-Body Skills Group component.

Institutional Review Board approval was obtained in 2018 to evaluate the impact of the Mind-Body Skills Groups. Simultaneously, given positive preliminary feedback from Mind-Body Skills Group participants in 2015–2017, the UAPD residency training directors added the course as an experiential component of residency didactic programming for all incoming postgraduate year 1 residents [14]. Thus, the participants in the Mind-Body Skills Group cohorts studied here include postgraduate year 1 residents and those participating in the Integrative Psychiatry Elective Track, namely, postgraduate year 3 and postgraduate year 4 residents and postgraduate year 5 and postgraduate year 6 fellows. Fellows from Child and Adolescent Psychiatry, Forensic Psychiatry, and Integrative Psychiatry took part.

The 10-week, 2-h-per-week Mind-Body Skills Group at the UAPD follows a model developed by The Center for Mind Body Medicine. Psychiatrist James S. Gordon created the Mind-Body Skills Group program and began using it with students at The Georgetown University School of Medicine in the mid-1990s [6]. Mind-Body Skills Groups teach the science and practice of evidence-based mind-body medicine approaches and techniques through experiential learning within a confidential small group setting [6]. The Mind-Body Skills Group at the UAPD was led by a Certified Facilitator for this model who was not in a supervisory role to the residents and fellows and was not a faculty or staff member of the UAPD.

The topics covered in the Mind-Body Skills Group at the UAPD followed the standard Center for Mind-Body Medicine model (see Table 1). Each 2-h session in the 10-week series follows a set pattern: opening meditation, check-in (sharing by participants in turn), didactic lesson, experiential skill training, check-out, discussion of home practice, and closing meditation. Participants agree to a set of group guidelines: confidentiality, mutual respect, commitment, practice, and the “I pass” rule, which allows them to refrain from speaking. Attendance and punctual arrival at each session were expected of all participants. Completing the course required missing no more than 2 sessions.

**Table 1:** Mind-Body Skills Group content

Week	Content
1	Introduction to mind-body medicine, breath regulation, and the use of simple drawings that promote awareness and expression
2	Biological underpinnings of mind-body medicine, biofeedback, and autogenics training
3	Guided imagery
4	Meditation practices including concentrative, mindfulness, and dynamic/expressive (movement-based) techniques
5	Understanding and exploring emotions, expressive writing
6 and 7	Family history, epigenetics, and exploring a solution-focused personal genogram
8	Nutrition, relationship to food, and mindful eating
9	Personal spirituality, forgiveness, gratitude, and compassion practices
10	Use of drawings that promote awareness and expression, closing ceremony

As a result of COVID-19 restrictions, the 2020 Mind-Body Skills Group was held entirely online via Zoom. All previous iterations were in person. Tech guidelines for the online group that applied to the mutual respect ground rule were emphasized and included maintaining a quiet, confidential space; minimizing movement and distractions in one’s environment; remaining on camera; and demonstrating attention to and engagement with the process and the others in the group. The exact flow, topics, and standardized group model and structure remained the same.

After the group was finished, a survey was administered to Mind-Body Skills Group participants. Respondents rated material/content taught on a scale of 1 to 10 (1 = poor, 10 = excellent). A 5-point Likert scale was used to assess satisfaction with the course, likelihood of using mind-body skills learned with patients in the future, and likelihood of recommending the Mind-Body Skills Group to other residents and fellows. Participants were also asked how frequently they were using skills they learned in the group for their own self-care and how frequently they were using the skills with their patients, with possible responses of daily, 2–3 times per week, weekly, monthly, and never. A yes/no question asked whether participants felt comfortable sharing their personal experiences in the context of the group. All these questions were followed by the option for a qualitative response, and a space for open-ended comments was provided at the end of the survey.

Differences between the in-person groups and the online group for all the ordinal data were analyzed using the Mann-Whitney U test. Fisher’s exact test was used to analyze the dichotomous data for the question about comfort in sharing personal experiences. All data were analyzed using IBM® SPSS® Statistics (v 27).

## Results

Overall group attendance for all three cohorts was high at 92.9%. All 50 participants completed the group and 44 of 50 filled out the survey and consented to the use of their survey results.

Overall, 66% of respondents were very satisfied and 18% were satisfied with the Mind-Body Skills Group. The material/content taught was rated excellent by 55% of participants, with 98% rating it above average. Seventy-three percent indicated that they would be extremely likely and 23% said they would be likely to recommend the Mind-Body Skills Group to other residents and fellows. Eighty percent of participants reported using skills learned in the group for their own self-care at least weekly. When asked how frequently they were using mind-body skills with their patients, 61% responded weekly or more frequently. Fifty-five percent of participants indicated that they were extremely likely and 34% likely to use mind-body skills with patients in the future. Because the Mind-Body Skills Groups included the ongoing opportunity to share personal experiences and feelings, participants were asked whether they felt comfortable sharing personal experiences with the group. Only 2 of the 44 respondents indicated that they did not feel comfortable; 95% said they were comfortable disclosing personal experiences to the group. There were no statistically significant differences in the responses to any of the questions for the in-person versus the online groups (see Table 2).

**Table 2:** Quantitative results of a survey of 44 participants in the Mind-Body Skills Group at the University of Arizona College of Medicine-Tucson Psychiatry Department. Breakdown by years for comparison between in-person groups (2018–2019) and online group (2020)

	2018 and 2019 (n=28)	2020 (n=16)	Overall (n=44)	Median	*z* test statistic	*p* value
Please rate the following: material/content taught in group						
1—Poor	0	0	0	10.0 in-person 10.0 online	.349	.727
2	0	0	0			
3	1 (3.57%)	0	1 (2.27%)			
4	0	0	0			
5—Average	0	0	0			
6	1 (3.57%)	0	1 (2.27%)			
7	1 (3.57%)	1 (6.25%)	2 (4.55%)			
8	4 (14.29%)	2 (12.50%)	6 (13.63%)			
9	6 (21.43%)	4 (25.00%)	10 (22.73%)			
10—Excellent	15 (53.57%)	9 (56.25%)	24 (54.55%)			
How satisfied are you with the 10-week Mind Body Skills Group?						
0—Very dissatisfied	2 (7.14%)	1 (6.25%)	3 (6.81%)	4.0 in-person 4.0 online	1.059	.290
1—Dissatisfied	0	0	0			
2—Neutral	4 (14.29%)	0	4 (9.09%)			
3—Satisfied	5 (17.86%)	3 (18.75%)	8 (18.18%)			
4—Very satisfied	17 (60.71%)	12 (75%)	29 (65.90%)			
How often are you using the mind-body medicine skills that you learned in this group for your own self-care?						
Daily	3 (10.71%)	3 (18.75%)	6 (13.64%)	4.3 in-person 4.3 online	−.102	.919
2 to 3 times per week	9 (32.14%)	3 (18.75%)	12 (27.27%)			
Weekly	11 (39.29%)	6 (37.5%)	17 (38.64%)			
1—Monthly	2 (7.14%)	4 (25%)	6 (13.64%)			
0—Never	3 (10.71%)	0	3 (6.82%)			
How often are you using the mind-body medicine skills that you learned in this group with your patients?						
Daily	1 (3.57%)	0	1 (2.27%)	4.3 in-person 4.3 online	-.051	.959
2 to 3 times per week	4 (14.29%)	4 (25.00%)	8 (18.18%)			
Weekly	12 (42.86%)	6 (37.5%)	18 (40.91%)			
1—Monthly	8 (28.57%)	2 (12.5%)	10 (22.73%)			
0—Never	3 (10.71%)	4 (25.00%)	7 (15.91%)			
How likely are you to use the mind-body medicine skills that you learned in this group with your patients in the future?						
4—Extremely likely	14 (50%)	10 (62.5%)	24 (54.55%)	3.5 in-person 4.0 online	.860	.390
3—Likely	10 (35.71%)	5 (31.25%)	15 (34.09%)			
2—Neutral	3 (10.71%)	0	3 (6.82%)			
1—Unlikely	0	1 (6.25%)	1 (2.27%)			
0—Extremely unlikely	1 (3.57%)	0	1 (2.27%)			
Would you recommend the 10-week Mind Body Skills Group to other residents?						
4—Strongly recommend	20 (71.43%)	12 (75%)	32 (72.73%)	4.0 in-person 4.0 online	.377	.706
3—Likely recommend	6 (21.43%)	4 (25%)	10 (22.73%)			
2—Neutral	1 (3.57%)	0	1 (2.27%)			
1—Not likely to recommend	1 (3.57%)	0	1 (2.27%)			
0—Would discourage	0	0	0			
Did you feel comfortable sharing your personal experiences in the group?						
1—Yes	26 (92.86%)	16 (100%)	42 (95.45%)	--	--	.526
0—No	2 (7.69%)	0	2 (4.44%)			

Qualitative comments about the Mind-Body Skills Group in the free text space after each question and in the open-ended comments were overwhelmingly positive. Many participants indicated that the course proved helpful in both personal and professional contexts. They mentioned specific skills or techniques, such as body movement and breathing exercises, genogram work, biofeedback, drawing, and journaling, that they had adopted for their own self-care. Some participants commented that the skills learned in the Mind-Body Skills Group provided ways they found useful in attempting to cope with stress. A few remarked on the fact that the course gave them additional insight into both the evidence base for mind-body skills and accessible language to use when discussing them with others. Some expressed the wish to continue attending group sessions following the 10-week Mind-Body Skills Group, but at less frequent intervals. Others wished to deepen their understanding and requested supplemental reading materials.

The most common negative comments concerned scheduling and timing, with some indicating that the 2-h sessions were longer than preferred. A few said that they felt the group did not quite prepare them to use mind-body techniques with patients and that they wanted more in the way of education on how to teach these skills to others. Although some said they appreciated the check-ins at the beginning of each session, others remarked that this took too much time from the substantive portion of each session. A couple made comments that they did not personally find the course useful at that time in their lives.

## Discussion

Residents and fellows surveyed responded favorably to the incorporation of a Mind-Body Skills Group into residency training, and survey results indicate that the course positively influenced resident and fellow behavior with regard to the adoption of mind-body techniques. Although effects on burnout itself were not surveyed here, Mind-Body Skills Groups teach the type of skills and techniques that have been shown to reduce stress and promote resilience, suggesting that Mind-Body Skills Groups may help protect against or ameliorate burnout among physicians [1, 3–6]. These findings are important in the context of a global pandemic, which has contributed to the challenges of burnout and stress among healthcare professionals [15].

Completion of the course by all 50 eligible residents and fellows and very positive responses in the post-intervention survey indicates that the Mind-Body Skills Group was acceptable to residents and fellows and feasible to implement as part of resident and fellow training at UAPD. The main findings are that the Mind-Body Skills Group was effective in providing residents and fellows with techniques they used for their own self-care, provided tools that residents planned to use with patients in the future, and was positively received by the participants despite the desire for some schedule adjustments.

A common theme in the open-ended comments was the need for more time for the Mind-Body Skills Group or to allow it to continue for a longer period; booster sessions were also suggested as beneficial. Satisfaction with the Mind-Body Skills Group remained positive, with no significant difference in responses, after the switch to an online format in 2020, a finding consistent with prior research showing that online psychotherapeutic programs may be as effective as in-person courses [8]. An online format may also make it easier for residents and fellows to incorporate the group into their schedules. Offering Mind-Body Skills Groups online opens collaborative possibilities between institutions and may help address situations in which a Mind-Body Skills Group Certified Facilitator is not available on site.

The qualitative and open-ended comments from the survey suggest that the UAPD Mind-Body Skills Group participants found support among one another while exploring mind-body skills and their potential utility together. This outcome can counter a culture in which residents feel stressed, lonely, and devalued, and it can be a powerful antidote to the forces that contribute to burnout [16], a theme that appears in the research on the effects of mind-body skills programs on students [5, 11]. One cohort of the UAPD Mind-Body Skills Group continued meeting to explore mind-body skills after the conclusion of the course.

Despite their benefits, Mind-Body Skills Group courses are resource intensive, may be challenging to conduct [6], and require time and expertise to assess. In addition to highlighting the potential professional impact of Mind-Body Skills Groups, this study also yields insight into various strategies for the implementation of Mind-Body Skills Groups. Financial and administrative supports are necessary, as are institutional champions who can facilitate incorporation of the group into a training program [6]. Mind-Body Skills Groups require funds for faculty, facilitator, and/or program coordinator time. Administrative support for incorporating the intervention into the schedule for didactics, as proposed by Lonergan, Duchin, Fromson, and AhnAllen [17], is also key.

This pioneering research on the effects of Mind-Body Skills Groups among psychiatry residents and fellows does have some limitations. The study lacked a control group. There was no comparison of dosing options to explore differential effects of courses held over a longer or shorter period or with different session lengths. While some survey respondents mentioned particular techniques that they found helpful, the impact of individual intervention components was not assessed, which makes it difficult to determine which were most effective. The sample included postgraduate year 3 and postgraduate year 4 residents and postgraduate year 5 and postgraduate year 6 fellows who had elected to participate in the Integrative Psychiatry Track as well as postgraduate year 1s for whom the course was included in didactics at the beginning of their residency. This means participants varied in their familiarity with and prior interest in Integrative Medicine and mind-body approaches. Because the survey did not identify respondents by postgraduate year, it was not possible to compare differential results.

Even with these limitations, this study adds depth to the research on the potential impact of Mind-Body Skills Groups and insight into implementation strategies. Results reveal potential positive impacts of Mind-Body Skills Groups among psychiatry trainees and begin to sketch a picture of ways that such training can affect psychiatric practice.
